# A Case Report on Adult-Onset Still’s Disease Successfully Treated With Tocilizumab: A Brief Review on its Safety and Efficacy

**DOI:** 10.7759/cureus.10098

**Published:** 2020-08-28

**Authors:** Shivaraj Nagalli, Ashish Sharma, Nidhi Shankar Kikkeri, Nasef Sherif

**Affiliations:** 1 Internal Medicine, Brookwood Baptist Health, Yuma, USA; 2 Internal Medicine, Yuma Regional Medical Center, Yuma, USA; 3 Neurology, University of Alabama, Birmingham, USA

**Keywords:** fever, arthritis, rash, ferritin, tocilizumab, refractory, adult-onset still's disease, steroids

## Abstract

Adult-onset Still’s disease (AOSD) is a rare autoimmune disease of unknown etiology with systemic inflammatory manifestations consisting of a triad of fevers, evanescent skin rash, and arthritis. Although steroids are the first line of therapy, about 20%-30% of patients are refractory, intolerant, and or relapse during tapering or upon discontinuation of steroids. There are no clinical guidelines in treating such patients and treatment in these patients is challenging. Previously used biological agents have limited efficacy and hence there is a need for new therapies. Tocilizumab (TCZ), an interleukin (IL)-6 receptor antibody has been used with a clinical benefit and has shown to decrease the dose of steroids in patients with adult-onset still disease. The aim of this case report is to highlight the use of tocilizumab in relapsing and steroid intolerant cases of AOSD. The use of this drug in patients with AOSD is currently off-label. Randomized control studies can provide additional information that offers better visibility in treating AOSD patients who are steroid-resistant or intolerant. The rarity of disease possesses additional challenges in conducting these studies.

## Introduction

Adult-onset Still’s disease (AOSD) is a rare autoimmune disease of unknown etiology with systemic inflammatory manifestations. A triad of fever, rash, and arthritis is commonly seen in patients with AOSD [1,2]. Steroids are the first line of therapy. However, there is a lack of clinical guidelines in the management of steroid-resistant and relapsing patients. Here we report this rare case of AOSD who relapsed after initial steroid therapy but responded to well to an interleukin-6 (IL-6) receptor antibody, tocilizumab (TCZ). We also do a brief review of the available literature on the efficacy and safety of TCZ in patients with AOSD.

## Case presentation

**Figure 1:**
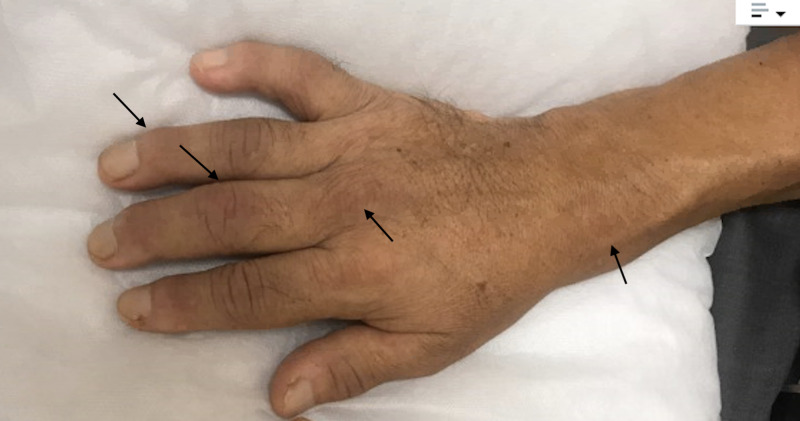
Dorsal surface of the right hand with swollen wrist, metacarpophalangeal, proximal, and distal interphalangeal joints as shown by the arrows.

**Figure 2:**
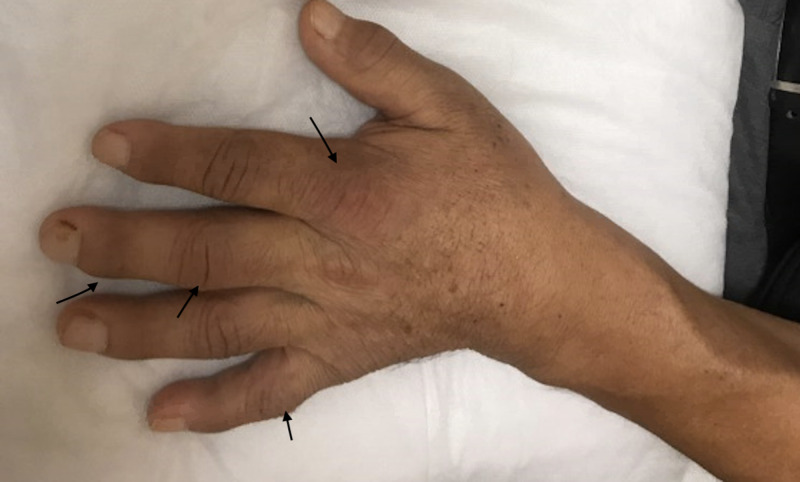
Dorsal surface of the left hand with swollen and inflamed metacarpophalangeal, proximal, and distal interphalangeal joints as shown by the arrows.

A 54-year-old male patient with a history of diabetes mellitus type II was admitted with complaints of pain and swelling of bilateral hands for two weeks. A review of systems was positive for intermittent fevers and fatigue. Physical examination was remarkable for synovial inflammation involving bilateral wrists, metacarpophalangeal, and proximal and distal interphalangeal joints as shown in Figures 1, 2 below.

A detailed skin evaluation was negative for rash. Initial laboratory studies were significant for leukocytosis of 15.7 K/microL (normal: 4.8-10.8 K/microL) with 86.5% of neutrophils (normal: 50%-70%) and 9% of lymphocytes (normal: 25%-40%). Hemoglobin was 7.7 g/dL (normal: 14-18g/dL) with a hematocrit of 23.9% (normal: 40%-52%). Peripheral smear was microcytic and hypochromic. Urinalysis was unremarkable with no evidence of proteinuria or hematuria.

Further evaluation consisting of infectious workup was negative. Inflammatory markers were elevated with an erythrocyte sedimentation rate (ESR) of more than 140 mm/hr (normal: 0-10 mm/hr) and c-reactive protein (CRP) at 167 mg/L (normal: <10 mg/L). Additional anemia workup revealed low serum iron level of 23 μg/dL (normal: 65-176 μg/dL), low total iron-binding capacity (TIBC) at 167 μg/dL (normal: 261-462 μg/dL) with a saturation of 13.8%. The patient was diagnosed to have anemia of chronic inflammation. Serum ferritin was extremely elevated at 10000 ng/ml (normal: 12-300 ng/ml).

**Figure 3:**
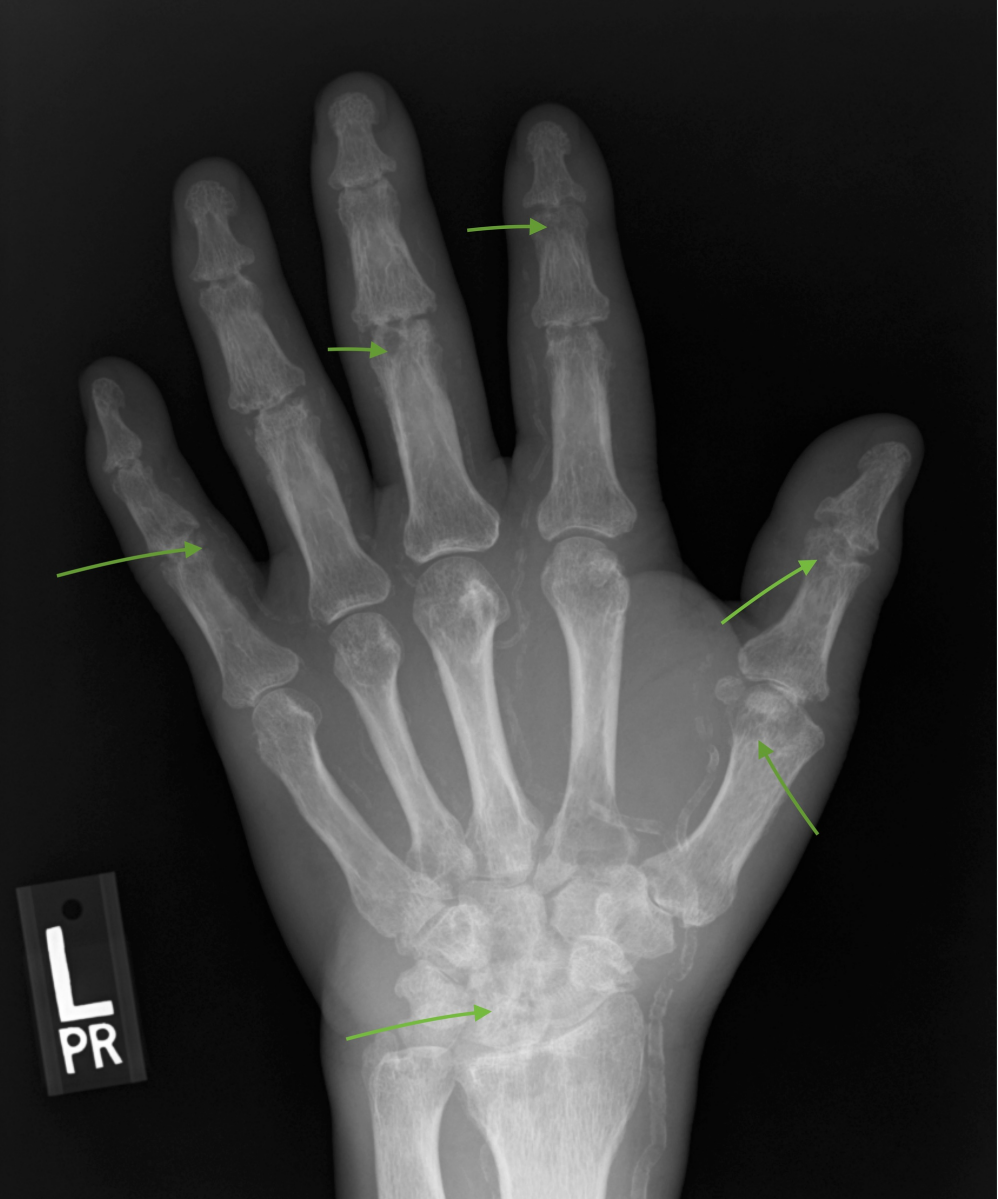
X-ray of the left hand showing joint erosions at the wrist, metacarpophalangeal, and interphalangeal joints as indicated by the arrows.

**Figure 4:**
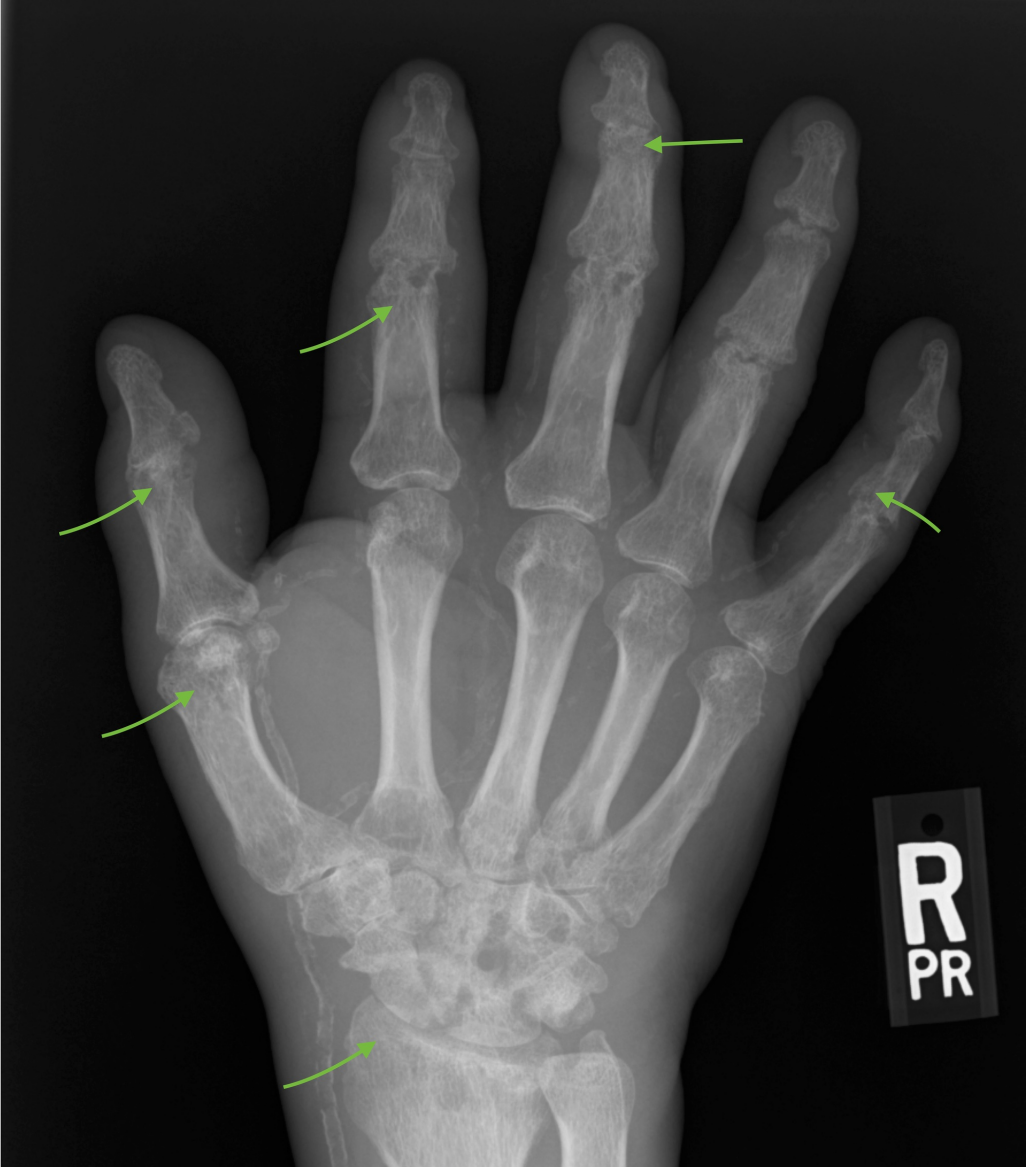
X-ray of the right hand showing multi-focal joint erosions at the wrist, metacarpophalangeal, and interphalangeal joints, as indicated by the arrows.

Radiological studies involving hand joints revealed multifocal joint erosions seen at bilateral radiocarpal, metacarpophalangeal, proximal, and distal interphalangeal joints with loss of joint spaces as shown in Figures 3, 4 below.

An autoimmune workup was subsequently done. The anti-nuclear antibody (ANA) was weakly positive at 1.6. Rheumatoid factor and anti-cyclic citrullinated peptide antibody (anti-CCP) were negative. Serological studies for systemic lupus erythematosus (SLE), Sjogren's disease were also negative. Complements were within normal limits. Human Immunodeficiency Virus (HIV), Hepatitis B, and Hepatitis C serology were non-reactive. CT of chest, abdomen, and pelvis revealed moderate pericardial effusion, mild cardiomegaly, and hepatosplenomegaly. However flow cytometry did not show evidence of monoclonal B cells, aberrant T cells, or blasts.

Owing to the presence of fever, leucocytosis, anemia of chronic inflammation with a negative infectious and hematological workup, a chronic inflammatory condition leading to polyarthritis was suspected. A negative rheumatoid factor and extremely elevated serum ferritin with levels > 10,000 ng/ml were suggestive of Adult-onset still’s disease. The patient was then started on oral prednisone with remarkable improvement in clinical symptoms and leucocytosis and was subsequently discharged.

About one-month follow up in the clinic serum ferritin levels had trended down to 1198 ng/ml. Hemochromatosis gene mutation was negative. Steroids were gradually withdrawn. Subsequent office visits showed recurrence of symptoms with worsening of inflammatory markers. Non-steroidal anti-inflammatory drugs (NSAIDs) and methotrexate were started with no clinical response and ESR and serum ferritin remained elevated more than 140 mm/hr and 10,000 ng/ml respectively. Steroids could not be restarted to treat AOSD relapse due to uncontrolled diabetes mellitus. Intravenous TCZ was infused at 8 mg/kg every four weeks were infused and monthly inflammatory markers were monitored.

Over the next few months, excellent clinical response was achieved with the complete resolution of the synovitis. Inflammatory markers trended down to ESR of 4 mm/hr and CRP was less than 1 mg/L. Ferritin was normalized at 338 ng/ml. Seven months after achieving remission, the patient continues to be symptom-free and is currently on the maintenance dose of TCZ.

## Discussion

AOSD is a rare autoimmune disease that was first described in 1971 [1]. It has slightly more predominant in females with a ratio of 1:1.3 and usual age of onset between 16 and 35 years; however, a late onset of the disease is also possible and well known [1]. Common symptoms include high spiking fevers (typically 39 degrees celsius or higher), evanescent rash, and polyarthritis [2]. Multiorgan involvement with lymphadenopathy, liver dysfunction, elevated ferritin levels, sore throat, and hepatosplenomegaly can also be seen [2,3]. The pathophysiology of this disease is poorly understood. Molecular studies have demonstrated a significant increase in the inflammatory cytokines like interleukins IL-1, IL-6, IL-18, tumor necrosis factor-α (TNF-α), interferon-γ (IFN-γ), indicating their critical role in the pathogenesis of AOSD [4-7].

NSAIDs and glucocorticoids have been the first line of therapy in AOSD. Disease-modifying anti-rheumatic drugs (DMARDs) have been used with some benefit. Agents like methotrexate, azathioprine, leflunomide, anakinra, and ciclosporin have been tried in the past but their effectiveness is limited [8-10]. There exists a small proportion of patients who respond poorly even to a higher dose of steroids and even when combined with other immunosuppressive agents and experience treatment-related complications. Treatment is challenging in these steroid-resistant patients and in patients who relapse during tapering or discontinuation of steroids which leads to the accrual of organ damage and long term side effects [11,12]. About 20%-30% of these patients do not respond to these traditional methods and get biological agents [13].

Several agents targeting the cytokines have been used for rescue to achieve remission [8,14-17]. TNF-alfa blockers like infliximab, adalimumab, and etanercept were the most commonly used biological agents in the past. However, they fell short in their effectiveness when compared to IL-1 and IL-6 inhibitors [18]. TCZ is a humanized IL-6 receptor monoclonal antibody with a favorable benefit in AOSD. Case reports, observational, and pilot studies have been done evaluating the safety and efficacy of TCZ. A systemic review on the published cases of AOSD treated with TCZ was done by de Boysson et al. and found that 30 out of 35 patients (86%) achieved a prompt articular improvement and a disappearance of systemic symptoms was seen in 27/28 (96 %) [19]. About 80% of the patients in the study were able to decrease steroid therapy and 20% of them were able to discontinue them. A small pilot study in 2017 by Li et al. involving a small sample of eight patients found that TCZ could alleviate the clinical manifestations of refractory AOSD rapidly and efficiently [4]. The study also demonstrated that the average dose prednisone was decreased to 17.1 mg/day from 51.3 mg/day (with over 30% reduction) at three months into therapy and the dose was further reduced to 11.7 mg/day at six months into the therapy.

The first and the only randomized double-blind placebo-controlled study by Kaneko et al. involving 27 patients found that TCZ is effective in improving patient’s symptoms at four weeks and in improving systemic symptoms and decreasing the dose of glucocorticoids [3]. 61.5% of the patients in the TCZ group and 30.8% (95% CI 9.1 to 61.4) in the placebo group achieved the primary outcome of ACR50 (American College of Rheumatology) response at four weeks. ACR50 was defined as a 50% improvement in the number of tender and swollen joints and improvement in at least three out of five predefined variable core sets. However, the ‘p’ value was 0.24 and the study did not achieve a statistical significance [3]. Nonetheless, at week 12, the placebo group participants were switched to receive TCZ and the ACR 50 response improved from 30.8% at week twelve to approximately 85% at week 16 which continued to maintain at one-year follow up [3]. Based on this study, in May 2019 the drug actemra (brand name for TCZ) was granted additional regulatory approval in Japan for its use in patients with AOSD who have not responded sufficiently to existing therapies.

A multi-center comparative study of TCZ with anakinra in patients with refractory to conventional treatment showed TCZ is more efficacious than anakinra [20]. Although the patients in both groups showed similar improvement in clinical parameters, the ESR and CRP levels improved faster in the TCZ group. Further, anakinra had to be discontinued in 11 patients (p=0.001) due to inefficacy [20]. Another observational study by Wang, et al. included patients with AOSD who have been refractory to steroids and another traditional immunosuppressive agent. Twenty-eight such patients reported significant improvement in clinical symptoms and inflammatory markers at eight weeks when TCZ (intravenous 8mg/kg) was given in addition to methotrexate (oral 12.5 mg once per week) [14]. This improvement sustained even after 48 weeks into the treatment. The dose of oral prednisone was decreased from 71.4 ± 20.7 mg/day to 3.3 ± 2.1 mg/day after a 48-week treatment (p<0.05) [14].

All the above studies suggest that TCZ is generally well tolerated and possesses a good safety profile. Overall, several case reports and observational studies have demonstrated the use of TCZ in the treatment of AOSD. Additionally, TCZ can contribute to a significant decrease in the dose of steroids and their associated side-effects. However adverse events have also been reported. In the above randomized controlled study by Kaneko et al. the most common side effect observed was nasopharyngitis. A few of the serious adverse effects attributed to TCZ in the study were subcutaneous abscess, cellulitis, pneumonia, splenic abscess, and anaphylactic shock [3]. Uncommon manifestations such as macrophage activation syndrome, indicated by coagulation abnormalities, decreased fibrinogen and hepatic dysfunction [4], organ failure and cytomegalovirus reactivation have also been reported and hence need close vigilance.

## Conclusions

There is a need for new therapies in managing patients with AOSD who are refractory to traditional and conventional treatments. Over the last several years, tocilizumab has continued to show favorable benefits in such patients and the data is promising. Besides, our case demonstrates its use in patients who cannot tolerate steroids and can be used as a steroid-sparing agent. However, not many randomized clinical trials exist to validate these results statistically and its use in the United States is currently off-label. Randomized control trials may be difficult due to the rarity of the disease and heterogenous nature of disease pattern but a multicenter collaboration can offer better visibility in treating steroid-resistant or intolerant patients.
